# Strategies to circumvent the CFTR defect in cystic fibrosis

**DOI:** 10.3389/fphar.2013.00108

**Published:** 2013-08-29

**Authors:** Frédéric Becq, Marc Chanson

**Affiliations:** ^1^Centre National de la Recherche Scientifique, Institut de Physiologie et Biologie Cellulaires, Université de PoitiersPoitiers, France; ^2^Laboratory of Clinical Investigation III, Department of Pediatrics,Geneva University Hospitals and University of GenevaGeneva, Switzerland

**Keywords:** CFTR chloride channel, CF, lung diseases, correctors, channelopathies

Mutations within the gene encoding for the chloride ion channel CFTR results in cystic fibrosis, the most common autosomal recessive genetic disease in the Caucasian population. CFTR regulates absorption and secretion mechanisms across intestinal and airway mucosae. Although the intestinal phenotype can be clinically handled, chronic infection and inflammation of the lungs of CF patients remains the principal cause of morbidity and mortality. The aim of this Research Topic is to provide to the readers the most recent information available on “Strategies to circumvent the CFTR defect in cystic fibrosis.” The Research Topic is divided in three mains parts: the first part describes the CFTR structure, processing and regulation of the normal and mutant ion channels. Anna Patrick and Philip Thomas (University of Texas Southwestern Medical Center, Dallas, USA) review the molecular interactions leading to the complex folding process of the CFTR protein and how essential steps are disrupted in CFTR mutants. Soo Jung Kim and William Skach (Oregon Health and Science University, Portland, USA) detail the timing and coordination of specific folding steps of the native CFTR in and across the ER membrane. Colleen Weiler and Mitchell Drumm (Case Western Reserve University, Cleveland, USA) broaden our views with genetic studies that have identified variant genes implicated in the clinical manifestations of CF. The second part of the Special topics discusses the molecular targets to rescue CFTR processing. Rebecca Chanoux and Ronald Rubenstein describe the current knowledge of the network of cellular chaperones that facilitate the folding and trafficking of CFTR to the plasma membrane. Then, a series of papers by the groups of Teresinha Leal (Université Catholique de Louvain, Brussels, Belgium) and John Hanrahan (McGill University, Montreal, Canada) report interesting observations regarding the potential of several inhibitors in correcting the CF phenotype at the level of ion transport and inflammation. Luigi Maiuri and collaborators (European Institute for Research in Cystic Fibrosis, San Raffaele Scientific Institute, Milan, Italy) propose to target the intracellular environment in order to reestablish functional autophagy in CF epithelial cells. The third chapter is dedicated to new molecules that have already been developed or in development and able to rescue CFTR channel function by targeting mutant CFTR at the mRNA (Michael Wilschanski, Hadassah Hospitals- Hebrew University, Jerusalem, Israel) and/or at the protein (Nicoletta Pedemonte and Luis Galietta, Laboratorio di Genetica Molecolare, Istituto Giannina Gaslini Genova, Italy) levels. Finally, the Special Topic is concluded by an in-depth review by Christine Bear and collaborators (The Hospital for Sick Children, Toronto, Canada). Importantly, the gaps in our knowledge regarding the mechanism of action of existing correctors, the unmet need to discover compounds which restore proper CFTR structure and function in CF affected tissues and new strategies for therapy development are discussed. We are convinced that we achieved a very interesting Special Topics thanks to the outstanding contributions of all authors. We are especially grateful to the authors for having believed in this project and accepted to share their knowledge. All the manuscripts have been peer-reviewed and we would like to thank the experts for helping us to reach an issue of high standard.

